# Natural Flavonoids for the Prevention of Sarcopenia: Therapeutic Potential and Mechanisms

**DOI:** 10.3390/ijms26157458

**Published:** 2025-08-01

**Authors:** Ye Eun Yoon, Seong Hun Ju, Yebean Kim, Sung-Joon Lee

**Affiliations:** 1 Department of Biotechnology, Graduate School of Biotechnology, Korea University, Seoul 02841, Republic of Korea; hinaprism@korea.ac.kr (S.H.J.); lastyb@korea.ac.kr (Y.K.); 2 Interdisciplinary Program in Precision Public Health, BK21 Four Institute of Precision Public Health, Korea University, Seoul 02841, Republic of Korea; 3 Department of Food Bioscience & Technology, Korea University, Seoul 02841, Republic of Korea

**Keywords:** sarcopenia, aging, muscle-atrophy, natural compounds

## Abstract

Sarcopenia, characterized by progressive skeletal muscle loss and functional decline, represents a major public heath challenge in aging populations. Despite increasing awareness, current management strategies—primarily resistance exercise and nutritional support—remain limited by accessibility, adherence, and inconsistent outcomes. This underscores the urgent need for novel, effective, and scalable therapeutics. Flavonoids, a diverse class of plant-derived polyphenolic compounds, have attracted attention for their muti-targeted biological activities, including anti-inflammatory, antioxidant, metabolic, and myogenic effects. This review aims to evaluate the anti-sarcopenic potential of selected flavonoids—quercetin, rutin, kaempferol glycosides, baicalin, genkwanin, isoschaftoside, naringin, eriocitrin, and puerarin—based on recent preclinical findings and mechanistic insights. These compounds modulate key pathways involved in muscle homeostasis, such as NF-κB and Nrf2 signaling, AMPK and PI3K/Akt activation, mitochondrial biogenesis, proteosomal degradation, and satellite cell function. Importantly, since muscle wasting also features prominently in cancer cachexia—a distinct but overlapping syndrome—understanding flavonoid action may offer broader therapeutic relevance. By targeting shared molecular axes, flavonoids may provide a promising, biologically grounded approach to mitigating sarcopenia and the related muscle-wasting conditions. Further translational studies and clinical trials are warranted to assess their efficacy and safety in human populations.

## 1. Introduction

Sarcopenia, an age-related loss of skeletal muscle mass and function, is a growing public health concern due to its impact on mobility, independence, and overall quality of life in aging populations. It leads to various negative health effects, such as extended hospital stays, increased risk of malnutrition, falls, fractures, and higher mortality [1]. The global prevalence of sarcopenia ranges from 10 to 27% among the elderly aged over 60 years and is expected to increase with the growing burden of aging populations [2,3]. Recognizing its clinical burden, the World Health Organization (WHO) and the United States assigned an individual international statistical classification of diseases and related health problems code for sarcopenia in 2016 (M62.84), prompting many other countries to follow suit. South Korea also adopted the sarcopenia code (KCD-8, M62.5) in 2021, facilitating epidemiological surveillance and reimbursement discussions. Despite the growing recognition of sarcopenia, no approved pharmacological treatment currently exists, and the development pipeline remains limited. As of 2024, several investigational agents are under preclinical or early-phase clinical trials targeting pathways such as myostatin inhibition, androgen receptor modulation, and mitochondrial biogenesis [4], but most have yet to demonstrate consistent efficacy or reach regulatory approval. 

According to the European Working Group on Sarcopenia in Older People 2 (EWGSOP2), sarcopenia is diagnosed based on low muscle strength, low muscle quantity or quality, and low physical performance. These diagnostic criteria provide a clinical framework to evaluate the progression and severity of sarcopenia in elderly populations [5]. However, given the ethnic differences in body composition and functional performance, the Asian Working Group for Sarcopenia (AWGS) has established diagnostic criteria specifically for Asian populations since 2014, with updates released in 2019 [6]. The AWGS emphasizes lower cutoff values for muscle mass and strength, reflecting the physiological norms of Asian cohorts. This distinction is akin to the difference between the Asian and European body mass index (a measure of body fat based on height and weight; BMI) classification standards. Thus, for sarcopenia research and clinical application in Asia, the AWGS provides a more regionally appropriate framework.

There are various confounding risk factors for sarcopenia. While a higher BMI may initially appear protective, this can reflect increased muscle mass; however, once adjusted for muscle quantity, both a higher BMI and greater visceral fat are associated with an elevated sarcopenia risk, indicating that excess fat alone is not protective [7,8,9]. Sarcopenic obesity, affecting approximately 11% of older adults (>60 years old) globally, is linked to poor outcomes [10]. Lifestyle factors such as physical activity and good nutrition are protective [11,12], whereas smoking and abnormal sleep duration increase the risk. Comorbidities including diabetes, osteoporosis, cardiovascular disease, cognitive impairment, respiratory dysfunction, depression, anorexia, and Parkinson’s disease are associated with increased sarcopenia risk [8]. Although the causal relationships remain unclear, proposed mechanisms include chronic inflammation, oxidative stress, insulin resistance, and mitochondrial dysfunction.

Chronic low-grade inflammation, or “inflammaging,” plays a key role in the pathogenesis of sarcopenia. Aging is associated with elevated systemic levels of pro-inflammatory cytokines, including TNF-α, IL-6, and C-reactive protein (CRP). These cytokines impair muscle anabolism by inhibiting the IGF-1/PI3K/Akt/mTOR pathway, which is critical for muscle protein synthesis [13]. Simultaneously, they activate catabolic pathways such as the ubiquitin–proteasome system, leading to the degradation of myofibrillar proteins [13]. For example, IL-6 has been shown to induce muscle atrophy by upregulating Atrogin-1 and MuRF1, two E3 ubiquitin ligases involved in muscle breakdown [14]. TNF-α exacerbates this process by activating NF-κB signaling, which promotes muscle wasting and suppresses regeneration [14]. Interestingly, although IL-6 is associated with chronic inflammation, it also functions as a myokine released by skeletal muscle during exercise. Under such conditions, IL-6 exerts anti-inflammatory effects by inducing Il-10 and inhibiting TNF-α expression [15]. Additionally, IL-6 enhances insulin sensitivity by activating AMPK signaling, promoting glucose uptake and fatty acid oxidation in muscle tissue, which may support metabolic health and muscle maintenance [16]. These beneficial actions of IL-6 suggest that its role in sarcopenia may be context-dependent and temporally regulated.

**Figure 1:**
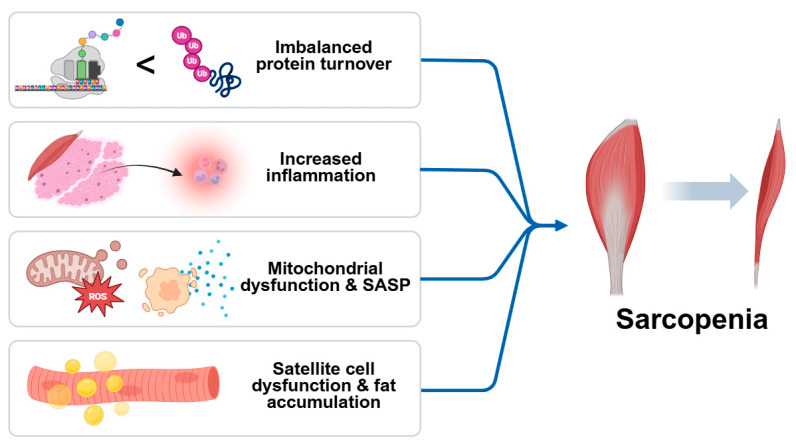
Schematic diagram of causes of sarcopenia. Sarcopenia is driven by multiple interrelated processes, including imbalanced protein turnover, chronic low-grade inflammation, mitochondrial dysfunction with increased ROS production, and satellite cell exhaustion. The accumulation of senescent cells exacerbates tissue damage via the senescence-associated secretory phenotype (SASP). Additional contributors include fat infiltration into muscle and the degradation of neuromuscular junctions, ultimately leading to progressive muscle mass and functional decline.

In addition to systemic inflammation, sarcopenia involves a convergence of the cellular and molecular mechanisms that contribute to progressive muscle loss. Central among these is an imbalance in protein turnover, where anabolic signaling is diminished while catabolic pathways are upregulated. Impaired IGF-1/Akt/mTOR signaling reduces protein synthesis, while activation of the ubiquitin–proteosome and autophagy–lysosome systems accelerates proteolysis. Chronic inflammation not only disrupts protein homeostasis but also compromises the function of satellite cells, limiting the regenerative capacity of skeletal muscle. Pro-inflammatory cytokines inhibit myogenic differentiation and promote fibrosis, resulting in a structural deterioration of muscle tissue [17]. Simultaneously, mitochondrial dysfunction emerges as a key contributor to sarcopenia by reducing energy availability and increasing reactive oxygen species (ROS) production. Excess ROS leads to oxidative damage of cellular components and activates redox-sensitive inflammatory pathways, creating a feedback loop that exacerbates muscle degeneration. The accumulation of senescent cells in aged muscle further amplifies local inflammation through the senescence-associated secretory phenotype (SASP), releasing cytokines, chemokines, and proteases that impair muscle function [18]. Moreover, aging muscle is prone to fat infiltration (myosteatosis), which alters muscle composition and insulin sensitivity, fostering a pro-inflammatory and metabolically compromised environment. The degradation of neuromuscular junctions also impairs motor unit recruitment, contributing to reduced muscle strength and coordination. These interlinked mechanisms—imbalanced protein turnover, chronic inflammation, oxidative stress, mitochondrial dysfunction, satellite cell exhaustion, and ectopic fat accumulation—collectively drive the progression of sarcopenia and underscore the need for comprehensive therapeutic strategies (Figure 1) [19].

Given the multifactorial etiology of sarcopenia, recent therapeutic strategies have primarily focused on resistance exercise, protein, amino acids, vitamins supplementation, and pharmacologic agents targeting anabolic pathways [20,21]. However, these interventions often face limitations in adherence, accessibility, and efficacy, especially among older adults with comorbidities or limited mobility. As a result, there is increasing interest in identifying natural, safe, and cost-effective alternatives capable of modulating the key biological processes involved in sarcopenia.

Among natural compounds, flavonoids—a large class of polyphenolic compounds found in fruits, vegetables, and medicinal plants—have attracted growing attention for their anti-inflammatory, antioxidant, anti-apoptotic, and metabolic-regulating properties. These bioactivities are particularly relevant to the pathogenesis of sarcopenia, which involves chronic inflammation, oxidative stress, and mitochondrial dysfunction. Preclinical and emerging clinical studies have shown that specific flavonoids such as quercetin, kaempferol, rutin, and catechins can protect against muscle atrophy, enhance mitochondrial biogenesis, improve insulin sensitivity, and stimulate muscle regeneration [14,15,16].

Moreover, flavonoid glycosides and their metabolites demonstrate improved bioavailability and tissue-specific targeting, enhancing their therapeutic potential. This review aims to summarize and critically evaluate the current evidence on nine natural flavonoids (quercetin, kaempferol, rutin, baicalin, genkwanin, isoschaftoside, naringin, eriocitrin, and puerarin) with anti-sarcopenic properties, focusing on their potential molecular mechanisms, experimental efficacy, and future clinical applicability. By organizing these compounds according to flavonoid subclasses, we aim to provide a comprehensive understanding of their functional roles in skeletal muscle preservation and regeneration.

## 2. Anti-Sarcopenic Effects of Natural Compounds

This section deals with the activity of the flavonoids against sarcopenia. Table 1 shows the model used in each experiment, the effects of the compounds, and molecular targets (Table 1).

### 2.1. Quercetin

Quercetin(3,3′,4′,5,7-pentahydroxyflavone) is one of the most studied polyphenolic flavonoids in the field of aging and age-related diseases. In particular, quercetin is one of the most frequently investigated compounds in sarcopenia research due to its potent antioxidant and anti-inflammatory properties. Quercetin is found in vegetables, fruits, tea and wine [55]. Quercetin is known to protect against aging through its antioxidant effects [56]. Due to its antioxidant effects inhibiting inflammatory receptors and their signaling pathway, these findings suggest that quercetin may prevent skeletal muscle inflammation and sarcopenia. In addition, further studies have been conducted on the efficacy of quercetin in suppressing sarcopenic obesity induced by sarcopenia or the de novo synthesizing of muscles by an anabolic pathway. Several studies investigated the fact that supplementation with quercetin reduces skeletal muscle atrophy and sarcopenia in vitro, in vivo, and in clinical trials.

Inflammatory cytokines such as NF-κB [23,57], TNFα [58,59], ERK, p38, and MAPK are known to major inflammatory members and are closely connected [22,60]. These inflammatory cytokines are upregulated under obese conditions and promote protein degradation [59]. Studies revealed that muscle atrophy is caused by inflammation and oxidative stress [61,62,63], which also suggests that the inflammatory response through the TNFα signaling pathways caused by obesity induces a loss of skeletal muscle.

When C57BL/6 mice were fed a high fat diet with 0.05% or 1% quercetin for nine weeks, the expression of inflammatory molecules (ERK, p38, MAPK and NF-κB) and macrophage accumulation were inhibited in the myotubes of the C57BL/6 mice [22]. The mRNA and protein levels of atrophic markers such as Atrogin-1 and MuRF1 were also reduced, rescuing the reduction of muscle mass and muscle fiber size. The reduction of inflammatory molecules and atrophic markers was also confirmed in C2C12 cells as well [22].

Kim et al. investigated the antioxidant effects of quercetin on TNFα-induced skeletal muscle atrophy in C2C12 cells [23]. Atrogin-1 and MuRF1 decreased in a concentration-dependent manner and an enhanced heme oxygenase-1 (HO-1) protein level was observed, which led to an increased nuclear translocation of Nrf2 in C2C12 cells. The inhibited atrophic responses were also confirmed in in vivo experiments with high-fat diet-induced obese mice. Quercetin supplementation upregulated Nrf2 and HO-1, leading to the inactivation of NF-κB. The quercetin actions were attenuated in the Nrf2-deficient mice [23]. Atrogin-1 and MuRF1 are components of the signaling pathway involving Akt and Foxo1 [64], and their expression level is regulated by the expressions of TNFα [65], ERK, p38, MAPK, and NF-κB [66,67].

Otsuka et al. aimed to assess the inhibiting effects of the administration of quercetin glycosides (QGs) on muscle atrophy in BALB/cCrSlc mice [24]. QGs were selected instead of quercetin aglycone due to their superior water solubility and bioavailability. Unlike aglycone forms, QGs are more efficiently absorbed in the small intestine after enzymatic hydrolysis by intestinal β-glucosidases, leading to increased systemic exposure to quercetin. Previous studies have demonstrated that quercetin glycosides exhibit greater absorption and plasma concentration compared to quercetin aglycone in both animal and human models [68,69,70]. Oral administration of QGs increased the weight of gastrocnemius muscle, which was decreased by dexamethasone. The mRNA levels of muscle atrophy-related genes including atrogin-1, MuRF-1, Foxo1, and myostatin that were elevated by dexamethasone treatment significantly decreased after QG treatment. The effect of quercetin itself was confirmed in murine C2C12 myotubes. Quercetin elevated the phosphorylation of Akt, which are downstream of the myostatin pathway, as well as the expressions of atrogin-1 and MuRF-1. These findings demonstrate that the protective effect of QGs on dexamethasone-induced muscle atrophy might depend on the suppression of myostatin signaling [24].

Muscle satellite cells (mSCs) are key resident stem cells responsible for the regeneration and preservation of muscle mass and function in adult skeletal muscle [71]. These cells, named for their distinct anatomical location, reside beneath the basal lamina in a predominantly quiescent state and are characterized by the expression of Pax7, a transcription factor belonging to the paired box (PAX) family. Upon activation, mSCs re-enter the cell cycle and begin expressing MyoD, a critical regulator of myogenic commitment, thereby transitioning into myoblasts. As proliferation continues, most myoblasts suppress Pax7 expression and initiate terminal differentiation via the upregulation of myogenin, ultimately leading to their fusion either with one another or with preexisting myotubes. However, with aging or metabolic stress, the regenerative capacity of mSCs declines, leading to impaired myogenesis and increased fat infiltration within skeletal muscle. Several studies have shown that intra- and intermuscular fat accumulation negatively affects muscle function and strength, and is closely associated with muscle wasting [72,73]. In obese conditions, such fat accumulation accelerates muscle degradation and contributes to the development of sarcopenic obesity, a condition characterized by the coexistence of reduced muscle mass and increased adiposity [74,75,76]. Interestingly, muscle satellite cells have been identified as a potential source of ectopic adipocytes in muscle tissue, as they can transdifferentiate into adipogenic lineages under certain pathological conditions. This suggests that mSCs not only regulate muscle regeneration but may also actively contribute to intramuscular lipid accumulation, highlighting their dual role in the pathogenesis of sarcopenic obesity.

Several studies imply that fat content within skeletal muscle may influence muscle function and strength. Quercetin inhibited the adipogenesis of muscle satellite cells with primary cells from the limbs of male F344 rats (10–14 weeks of age) under adipogenic conditions. Quercetin (5 µM to 30 µM) suppressed triglyceride contents and the triglyceride-to-protein ratios in muscle satellite cells in a dose-dependent manner. Moreover, the upregulation of mRNA levels for adipogenic markers (PPAR-γ and FABP4) was suppressed by administering quercetin at concentrations ranging from 5 µM to 50 µM for 6 days in primary mSCs [25]. Additionally, quercetin attenuated lipid accumulation in human muscle-derived PDGFRα+/CD201+ cells under adipocyte differentiation conditions. These cells are a population of mesenchymal progenitors residing in skeletal muscle, known to contribute to ectopic fat formation under pathological conditions. Attenuated adipogenic gene expression (CEBPA and ADIPOQ) and lipid accumulation were employed via the inhibition of CREB phosphorylation [26]. Moreover, quercetin prolonged the exhaustive swimming time in male BALB/c mice by increasing the mRNA expression of fatty acid β-oxidation-related genes such as PPAR-δ, CPT1, β-hydroxyacyl coenzymes A dehydrogenase, and UCP3 in the skeletal muscle of mice [27].

In summary, quercetin shows a preventive effect against sarcopenia by suppressing lipid accumulation within skeletal muscle. Similarly, sarcopenic obesity may be associated with increased inflammatory responses. Therefore, further research should be conducted to elucidate the relationship between lipid accumulation and inflammatory reactions within skeletal muscle in a state of obesity.

### 2.2. Rutin

Rutin (quercetin-3-O-rutinoside) is a glycoside of quercetin composed of quercetin and rutinose [77,78]. Rutin content is abundant in foods such as buckwheat, peppers, apples, cherries, and aronia [79,80]. It is noteworthy that rutin has been shown to be effective in its antioxidant and anti-inflammatory functions in in vitro and in vivo experiments [81,82,83]. In addition, rutin modulates cell differentiation, proliferation, and metabolism by the PI3K and mTOR pathways [84,85]. The findings imply that rutin has therapeutic potential due to its antioxidant and anti-inflammatory properties, and it also modulates anabolic processes in muscle. In other words, rutin protects muscles from damage and maintains homeostasis, thereby potentially preventing sarcopenia.

Rutin plays a crucial role in muscle synthesis and tissue composition. Reduced glucose consumption leads to sarcopenia by antiatrophic agents such as glucocorticoid [86]. Therefore, regulating glucose uptake is crucial for glucose consumption and subsequently enhancing muscle mass [87,88]. T-2 toxin alters protein expression, leading to abnormal muscle development. In shrimp muscle, rutin restored the altered protein expressions to normal levels. Specifically, it upregulated the expressions related to carbohydrate metabolism (enolase, malate dehydrogenase, and ATP/ADP translocase) and protein translation (elongation factor 1-alpha, eukaryotic translation initiation factor 2 subunit alpha, and aspartate aminotransferase) [28]. Obesity also causes sarcopenia because it is associated with metabolic disorders [89]. Recent studies revealed that rutin regulated metabolism by promoting glucose uptake through GLUT4 translocation in the soleus muscle of high-fat diet-induced obese ICR mice [29,30]. Additionally, the enhanced metabolism and the inhibition of fat accumulation involved the phosphorylation of AMPK, CAMKKβ, and ACC in adipocytes [29]. Kappel et al. conducted experiments with 50-day-old male Wistar rats to explain GLUT-4 translocation in skeletal muscle. Considering these experiments, rutin promoted GLUT-4 translocation through increased calcium signaling via the activation of CaMKII [31]. Moreover, high-fat diet-induced obese male Sprague-Dawley rats exhibited reduced mitochondrial content in skeletal muscle, but rutin increased their mitochondrial DNA content by AMPK activation. Similarly, in mice with diabetes induced by hyperglycemia, rutin modulates the expressions of Atrogin-1, MuRF-1, PGC-1α, Nrf-1, Bax, and Bcl-2. This regulation promotes mitochondrial biogenesis and inhibits muscle cell apoptosis. Additionally, rutin enhanced the expression of genes associated with mitochondrial biogenesis, including PGC-1α, NRF-1, Tfam, and SIRT1 [32]. Also, rutin is the polyphenol compound of castor oil plant leaf (*Ricinus communis* L., RC) extract and it increases the expression of myogenesis factors such as MyoD, MyoG, and MHC isoforms [33]. In conclusion, rutin promotes energy metabolism and muscle synthesis through the enhancement of mitochondrial biogenesis and glucose uptake, thereby preventing sarcopenia.

Studies also have applied the antioxidant and anti-inflammatory properties of rutin in muscle. The treatment of C2C12 myoblasts with rutin, following lipopolysaccharide (LPS)-induced inflammation, resulted in a reduction of NF-κB, detected by electrophoretic mobility shift assay. Subsequently, rutin decreased both mRNA expression and the secretion of IL-6, which is the downstream target of NF-κB [34]. Additionally, rutin restored the increased expressions of MAFbx, MuRF1, and FOXO3 proteins and induced senescence by dexamethasone in C2C12 cells [35]. Dietary supplementation of rutin mitigated the oxidative stress of the vascular smooth muscle cells of C57BL/6 mice using hydrogen peroxide-induced senescence and the breast muscle of Qingyuan partridge chickens and broilers. This protection was evidenced by a reduction in malondialdehyde and ROS levels, which contributed to the preservation of telomeres [36,37,38]. Additionally, the expression of oxidative stress markers such as 3-nitrotyrosine, 4-hydroxynonenal, p47phox, and Nox4 was also decreased [36]. Also, rutin conferred antioxidant benefits through a higher total antioxidant capacity and glutathione peroxidase activity. Furthermore, rutin inhibited the expression of LC3-II, associated with mitophagy, and blocked the PINK1/Parkin signaling pathway [38]. Recently, the dietary intake of rutin restored the muscle weight of the gastrocnemius, tibialis anterior, and extensor digitorum longus in mice with dexamethasone-induced sarcopenia. This effect is because of the inhibition of the FOXO3/MAFbx and FOXO3/MuRF1 pathways, which is related to ubiquitin–proteasome activation [35]. As mentioned earlier, rutin is a functional polyphenol compound of *Ricinus communis* L. [33,90,91]. It reduced the activity of the E3 ubiquitin ligases (Atrogin-1 and MuRF1) associated with muscle atrophy in C2C12 myotubes. Remarkably, this effect occurred regardless of the presence of dexamethasone which induces muscle atrophy. Moreover, rutin alleviated oxidative stress by scavenging 2,2-diphenyl-1-picrylhydrazyl and 2,2′-azino-bis(3-ethylbenzothiazoline-6-sulfonic acid) radicals [33]. Rutin is also present in both *Syringa oblata* Lindl. and *A. edulis* decoction extracts, suggesting an alleviation of excessive inflammatory responses in muscle [39,40,92]. These extracts commonly regulated the expression of SOD and catalase in mice experiencing induced aging with D-galactose and fed a high-fat diet [39,40]. Specifically, *Syringa oblata* Lindl. extract increased running ability and decreased the expression of MDA, BUN, lactate, GOT, GPT, nNOS, iNOS, TNF-α, and syncytin-1 in serum or muscle [39]. Furthermore, *A. edulis* extract inhibited the phosphorylation of IκB kinase (IKK) [40]. Overall, rutin protects against oxidative stress induced by inflammation or aging in myotubes and the muscles of various animals, thereby preventing muscle atrophy.

In summary, rutin enhances energy metabolism to promote muscle synthesis and protects against muscle loss through its antioxidant and anti-inflammatory properties, offering a potential solution for sarcopenia. Troxerutin, a trihydroxyethylated derivative of rutin, is more tolerable at high doses and has a higher safety profile in the digestive system [93]. In hereditary hypertriglyceridaemic rats, a 4-week diet containing troxerutin reduced triglyceride accumulation in skeletal muscle, improved insulin sensitivity, and increased glucose uptake [94]. Additionally, the sodium salt of rutin also has shown higher solubility and bioavailability [95]. Dietary intake of the sodium salt of rutin in C57BL/6 mice improved behavioral performance and reduced myocardial fibrosis and skeletal muscle atrophy, as observed through histopathological analysis [96].

One human trial study suggests that rutin, when administered as part of a bioactive nutritional supplement alongside whey protein and omega-3 fatty acids, may contribute to improve muscle function in elderly individuals with mobility limitations [97]. Specifically, the whey protein isolate + bioactive capsules group, which included rutin, showed a significant 13% increase in knee extension strength and the greatest improvement in gait speed (8%) compared to the control, whereas whey protein alone did not yield significant benefits [97]. These results highlight rutin’s potential role in mitigating age-related sarcopenia. However, further studies, including well-controlled human trials, are warranted to validate the efficacy and mechanistic contribution of rutin to sarcopenia prevention and treatment.

### 2.3. Kaempferol Glycosides

Kaempferol extracted from plants improves muscle function and induces differentiation through enhanced glucose absorption in muscle cells. Kaempferol 3-neohesperidoside, a kaempferol glycoside extracted from *Cyathea phalerata*, promoted glucose uptake and glycogen synthesis in rat soleus muscle [41], as effectively as insulin. Kaempferol-3-neohesperidoside has been shown to stimulate glycogen synthesis in rat soleus muscle via multiple signaling pathways [41]. The stimulatory effect was inhibited by wortmannin, a specific inhibitor of PI3K, and was further enhanced by lithium chloride, an inhibitor of GSK-3, indicating the involvement of the PI3K–GSK-3 axis. Additionally, the effect was abolished by PD98059, a MEK inhibitor, and by calyculin A, an inhibitor of PP1, suggesting a role for the MAPK–PP1 signaling pathway. These findings collectively suggest that kaempferol-3-neohesperidoside promotes glycogen synthesis through the coordinated activation of both the PI3K–GSK-3 and MAPK–PP1 pathways [41].

Kaempferol 3-O-rutinoside, extracted from *Antidesma acidum* Retz, was shown to enhance glucose uptake in L6 skeletal muscle cells with sodium palmitate-induced insulin resistance by promoting SIRT1 expression [42]. The induction of SIRT1 via kaempferol 3-O-rutinoside treatment upregulated p-IRS, AKT, AMPK signaling molecules, and GLUT4 translocation. This led to glucose uptake in L6 myotubes with sodium palmitate-induced insulin resistance. A molecular docking and simulation study showed a strong involvement of the interaction between the kaempferol part of kaempferol 3-O-rutinoside and SIRT1. These effects were attenuated in the SIRT1-knockdown myotubes [42].

Several studies have shown that the anti-inflammatory and antioxidant effects of kaempferol significantly inhibit muscle atrophy [43,98,99,100]. Molecular docking studies of kaempferol indicated its good binding ability with TGF-β1, COX-2, p53 (Tumor Protein 53), and RELA (v-rel avian reticuloendotheliosis viral oncogene homolog A) [98,101]. Namely, kaempferol, the major active compound of Astragalus membranaceus, demonstrates a therapeutic potential for regulating inflammation [98]. *Ricinus communis* L. contains kaempferol-3-O-β-D-xylopyranoside, kaempferol-3-O-β-D-glucopyranoside, and kaempferol-3-O-β-rutinoside [99]. These compounds alleviated muscle atrophy in C2C12 cells induced by dexamethasone by reducing mitochondrial oxidative stress [33]. Kaempferol also maintained a thicker and longer morphology of C2C12 cells during differentiation, reduced lipohydroperoxide concentration, and decreased ROS levels by modulating reactive oxygen and nitrogen species signaling [100]. Kaempferol is also present in the plant *K. pinnata* [43,102]. It increased catalase (CAT) activity and reduced malondialdehyde levels in human skeletal muscle myoblasts (HSMMs) and human diabetic skeletal muscle myoblasts (DHSMMs) in combination with metformin [43]. In broiler chickens, kaempferol hydroalcoholic extracts from *Cyathea phalerata* stems reduced thiobarbituric acid reactive substances (TBARSs) to normal levels in hypercholesterolemic muscle and inhibited the formation of malondialdehyde (MDA) [44]. Furthermore, cerebral palsy is related to decreased anabolic responses like that observed in elderly individuals with sarcopenia [103]. Notably, intraperitoneal injection of kaempferol (1 mg/kg) in neonatal Wistar rats with cerebral palsy reduced oxidative fibers and mitigated the decrease in the weight of the soleus muscle and the area of a cross-section of type I soleus muscle fibers [45]. Therefore, kaempferol inhibits sarcopenia by regulating oxidative stress in muscle, thereby exerting antioxidant and anti-inflammatory effects.

Taken together, kaempferol glycosides can alleviate sarcopenia not only by promoting glucose uptake and differentiation but also by regulating inflammation in muscle. Although kaempferol has potential biological effects, few clinical trials were found about a dosage of kaempferol glycosides [104,105]. Recently, a kaempferol aglycone supplement had no side effects in healthy participants up to a dosage of 50 mg, which was five times higher than the dietary intake of kaempferol [106,107]. Further clinical trials are required to clarify the effect of kaempferol and its aglycone in human muscle.

### 2.4. Baicalin

Baicalin (7-glucuronic acid 5,6-dihydroxyflavone), primarily derived from the ancient Chinese herbal medicine Radix Scutellariae, is known for its potent antioxidant, anti-inflammatory, and anticancer properties [108]. Baicalin has demonstrated significant anti-inflammatory activity in various in vitro and in vivo disease models by inhibiting pro-inflammatory cytokines and NF-κB activation [109,110,111,112,113,114]. NF-κB activation is associated with the upregulation of MuRF1 and MAFbx, key factors in muscle atrophy, and has been shown to cause severe muscle wasting in in vivo experiments [115,116,117,118]. Studies using knockout animals lacking MuRF1 or MAFbx have confirmed that these proteins contribute to skeletal muscle atrophy [117,118].

Cancer cachexia shares common pathological features with sarcopenia, including progressive skeletal muscle wasting and elevated inflammatory signaling. Due to these similarities, cachexia models are often used to investigate potential therapeutic agents for muscle atrophy. A cancer cachexia mouse model was established using CT-26 colon adenocarcinoma cells, and baicalin was administered intraperitoneally. The expressions of the MuRF1, MAFbx, and NF-κB pathway proteins were analyzed by Western blotting. The results showed that baicalin significantly alleviated muscle weight loss in the cancer cachexia mice and reduced the expressions of MuRF1 and MAFbx, along with inhibiting the activation of the NF-κB pathway [46]. This suggests that baicalin effectively reduces tumor-induced inflammatory responses and mitigates muscle atrophy.

### 2.5. Genkwanin

Genkwanin (5,4′-dihydroxy-7-methoxyflavone) is a flavone compound and isolated from plants such as *Daphne genkwa*, *Salvia officinalis* L., *Rosmarinus officinalis*, and *Equisetum arvense* [119,120,121]. Several studies revealed that the extracts from plants containing genkwanin exhibited antioxidant and anti-diabetic properties [122,123,124]. In particular, genkwanin has demonstrated beneficial effects in alleviating rheumatoid arthritis which is closely associated with sarcopenia [125,126,127]. Therefore, genkwanin holds potential as a research material targeting sarcopenia.

Several studies have demonstrated the effects of plants containing genkwanin on muscle cells. A 50 µg/mL concentration of *Aquilaria sinensis* flower extract (50 μg/mL) containing genkwanin increased glucose uptake on differentiated C2C12 myotubes by 32.8% in a GLUT4-dependent manner [47]. Recently, *Equisetum arvense* extract, also containing genkwanin, was analyzed by ultra-high performance liquid chromatography-tandem mass spectrometry (UHPLC-MS). It restored the reduced C2C12 myotube diameters induced by TNFα/IFNγ (T/I) or dexamethasone. Additionally, it counteracted protein degradation by increasing the expressions of MuRF-1 and Foxo3. In silico modeling also revealed its anti-inflammatory properties through the inhibition of NF-κB signaling. Furthermore, experiments with pre-geriatric C57BL/6 mice demonstrated that a dietary intake of *Equisetum arvense* extract at 500 mg/kg/day for three months resulted in the maintenance of muscle mass and performance [48]. To summarize, genkwanin has been revealed to possess functions that alleviate sarcopenia. Therefore, further studies about the effects of plant extracts containing genkwanin or the genkwanin compound itself on skeletal muscle need to be studied more.

### 2.6. Isoschaftoside

Isoschaftoside, a bioactive compound in wheat seedlings extract, has shown promise in combating muscle atrophy, particularly sarcopenia. The compound’s potential has been evaluated through various cellular and molecular experiments that focus on its anti-inflammatory and mitochondrial-supporting properties [49].

Isoschaftoside was tested on C2C12 myotubes, a model for skeletal muscle cells. Muscle atrophy was induced by exposing these cells to the tumor necrosis factor-alpha (TNFα), a cytokine that triggers inflammation and muscle degradation. The results showed that isoschaftoside treatment significantly reduced muscle atrophy. Specifically, the diameter of myotubes was preserved compared to the control group, indicating a protective effect against muscle wasting [49]. Isoschaftoside also had a notable impact on mitochondrial biogenesis and inflammation [49]. The mRNA and protein expression of myogenic regulatory factors such as Myf5, Myf6, MyoD, and Myogenin were significantly increased after isoschaftoside treatment in C2C12 cells. The mRNA expressions of TNFα, IL-1α, IL-1β, and IL-6 were reduced when compared to the TNFα-treated group. These findings suggest that isoschaftoside suppresses muscle atrophy by reducing inflammation and activating mitochondrial biosynthesis.

These experimental results indicate that isoschaftoside has a multifaceted protective effect against muscle atrophy, acting through the preservation of muscle cell structure, the promotion of muscle regeneration, and the support of mitochondrial function. These findings suggest that isoschaftoside could be a promising therapeutic agent for preventing or treating sarcopenia and other conditions related to muscle atrophy, especially in aging populations.

### 2.7. Naringin

Naringin, a flavanone glycoside predominantly found in citrus fruits, is well-known for its diverse pharmacological properties, including its antioxidant, anti-inflammatory, and anti-apoptotic effects. Its chemical structure is characterized by the presence of rhamnoglucoside moieties, which contribute to its bioactivity [50]. Due to these properties, naringin has been studied extensively for its potential health benefits, particularly in muscle health and the prevention of sarcopenia.

Naringin has been shown to improve muscle function and reduce fatigue. A study demonstrated that dietary supplementation with 0.04% naringin significantly prolonged the exhaustive swimming time of mice, indicating enhanced endurance. This improvement is attributed to naringin’s ability to enhance mitochondrial function and antioxidant capacity, which are crucial for sustaining muscle activity during prolonged exercise [50]. The antioxidant properties of naringin play a significant role in its muscle-protective effects. Naringin supplementation increased the activities of key antioxidant enzymes, including superoxide dismutase (SOD), catalase (CAT), and glutathione peroxidase (GSH-Px) in muscle tissues. These enzymes help in scavenging reactive oxygen species (ROS), thus preventing oxidative stress-induced muscle damage [50].

Glycogen serves as a critical energy reservoir for muscle contraction, especially during anaerobic or high-intensity activities, which predominantly rely on type II fibers. Age-related reductions in muscle glycogen content have been associated with impaired exercise tolerance, muscle fatigue, and metabolic inflexibility. Therefore, interventions that enhance glycogen accumulation or utilization may contribute to improved muscle performance and delay the functional decline associated with sarcopenia [128]. One of the mechanisms by which naringin enhances muscle function is by increasing glycogen storage in muscle tissues. Chen et al. showed that naringin supplementation significantly increased the glycogen content in both liver and muscle tissues. Glycogen is a crucial energy reserve that sustains prolonged muscle activity, thus enhancing endurance and reducing fatigue. Given its multifaceted benefits, naringin holds promise as a therapeutic agent for preventing and managing sarcopenia. Its ability to enhance muscle endurance, improve mitochondrial function, increase antioxidant capacity, and reduce inflammation makes it a potent candidate for muscle health supplementation [50].

Naringin also ameliorated skeletal muscle atrophy in obese rats. Treatment with naringin after inducing obesity through a high fat diet (HFD) significantly reduced body weight, and the levels of total cholesterol and triglycerol in muscle tissues when compared to the HFD group. The naringin-treated group also showed decreased levels of creatine kinase, lactate dehydrogenase, and aspartate transaminase in serum, indicating less muscle injury. Moreover, naringin significantly increased the mRNA expression of protein synthesis-related genes, such as mTOR and PGC1-α while decreasing the expression of protein degradation-related genes, including Atrogin-1 and MuRF-1, in quadricep tissues. This led to an increase in the muscle mass of soleus, gastrocnemius, and quadriceps [51].

In summary, naringin exhibits significant potential in improving muscle function and preventing sarcopenia through its antioxidant, anti-inflammatory, and muscle fiber-modulating effects. Future studies should focus on clinical trials to confirm these benefits in human subjects and explore optimal dosing strategies for maximum efficacy.

### 2.8. Eriocitrin

Eriocitrin, a flavonoid glycoside, is predominantly found in lemon peels (*Citrus limon*). Its chemical structure is characterized as 3′,4′,5,7-tetrahydroxyflavanone-7-[6-O-(α-L-rhamnopyranosyl)-β-D-glucopyranoside]. Eriocitrin exhibits potent antioxidative properties, which have been explored for various health benefits including the amelioration of metabolic disorders and muscle atrophy [129].

Sarcopenia is exacerbated by oxidative stress and inflammation. Eriocitrin has shown promising results in reducing these stressors in muscle tissues. In studies involving denervated mice, eriocitrin significantly suppressed the expression of muscle atrophy-related ubiquitin ligases, such as Atrogin-1 and MuRF-1, which are typically upregulated in response to increased reactive oxygen species (ROS) [52]. Eriocitrin intake prevented weight loss in the gastrocnemius muscle of denervated mice by downregulating the expression of these ubiquitin ligases. The antioxidative effect of eriocitrin was evident as it inhibited lipid peroxidation and maintained the glutathione disulfide/glutathione (GSSG/GSH) ratio. This suggests that eriocitrin helps in maintaining the redox balance within muscle tissues, thereby protecting them from oxidative damage [52].

Additionally, the suppression of oxidative stress markers, such as lipid peroxide (LOOH) and the GSSG/GSH ratio, was observed in mice treated with eriocitrin. This antioxidative defense mechanism is crucial in preventing the degradation of muscle proteins via the ubiquitin–proteasome pathway. Eriocitrin’s ability to modulate these pathways underscores its potential as a therapeutic agent against muscle atrophy and sarcopenia [130].

In summary, eriocitrin’s multifaceted role in reducing oxidative stress, improving mitochondrial function, and downregulating muscle atrophy-related genes makes it a promising candidate for combating sarcopenia. These findings suggest that dietary supplementation with eriocitrin could be beneficial for elderly individuals at risk of muscle degeneration.

### 2.9. Puerarin

Along with the long history of traditional Eastern Asian culture, the variability of bioactive ingredients has been a focal point of pharmacological research [131]. Puerarin, the principal bioactive ingredient from *Pueraria lobata* (Willd.) Ohwi (commonly known as “Ge gen”) [132], was originally isolated from its roots in the 1950s. In Korea, *Pueraria lobata* has also been traditionally used for centuries as a medicinal and dietary plant, particularly for treating a stroke [133].

There is substantial evidence supporting puerarin’s protective effects on muscle-related diseases. Puerarin treatment enhances mitochondrial biogenesis and oxidative phosphorylation while simultaneously reducing ectopic lipid accumulation [134,135]. By modulating the Akt/mTOR pathway and autophagy, puerarin prevents skeletal muscle wasting, leading to significant increases in skeletal muscle weight and strength in type 1 diabetic rats [53]. The regulation of energy metabolism in skeletal muscle under *Pueraria lobata* administration involves the activation of PGC-1α and AMPK. The recovery of palmitate-induced lipotoxicity in L6 myotubes further supports puerarin’s efficacy [54]. According to Xufeng Chen et al. [136], puerarin is also considered a potential therapy for sarcopenia. Rodent studies provide evidence that puerarin may alleviate skeletal muscle atrophy through autophagy [137] and neural regeneration [138]. Thus, it is plausible to speculate that puerarin can restore muscle atrophy as well as muscle weight and function.

Overall, the therapeutic potential of puerarin in skeletal muscle is promising according to the literature. Similar to other natural derivatives, the mechanisms by which puerarin exerts its beneficial effects on skeletal muscle are complicated. Nevertheless, considering the wide range of puerarin-mediated effects in various pathological conditions, it is noteworthy that puerarin may be suitable for modern patients suffering from malignancies. Therefore, to develop effective puerarin-derived therapies, further mechanistic studies are essential.

## 3. Discussion

**Figure 2:**
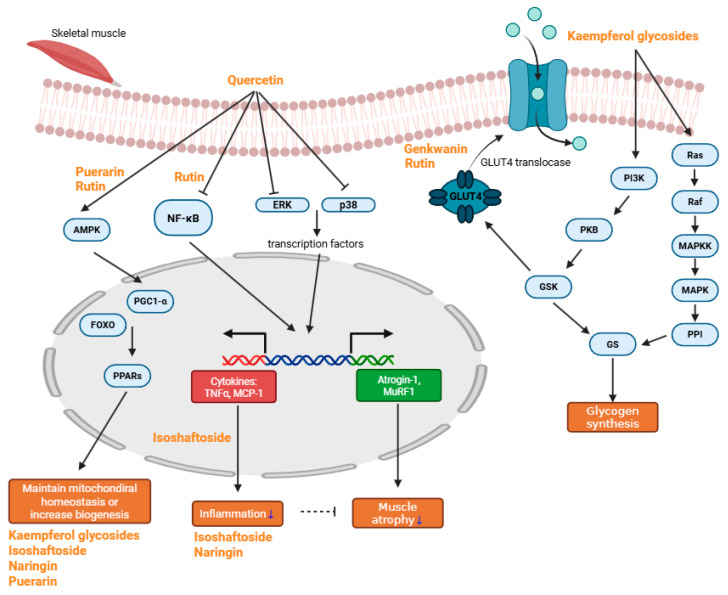
Major schematic pathway of anti-sarcopenic compounds. Quercetin reduces muscle atrophy and inflammation by inhibiting NF-κB, ERK, and p38, thereby suppressing the transcription of TNF-α, MCP-1, Atrogin-1, and MuRF1. It also helps maintain mitochondrial homeostasis by regulating AMPK, which subsequently activates PPARs. Rutin primarily alleviates inflammation through inhibition of the NF-κB pathway and promotes mitochondrial biogenesis via AMPK activation. Both rutin and genkwanin enhance glucose uptake and help prevent hyperglycemia. Kaempferol glycosides mainly modulate the PI3K/Ras pathway, thereby promoting glycogen synthesis. Some kaempferol glycosides, along with isoschaftoside, naringin, and puerarin, effectively preserve mitochondrial homeostasis or enhance mitochondrial biogenesis. Additionally, isoschaftoside and naringin exhibit anti-inflammatory effects (blue arrow indicates decrease in inflammation and muscle atrophy).

Sarcopenia, a multifactorial condition driven by inflammation, oxidative stress, mitochondrial dysfunction, and impaired muscle regeneration, demands multifaceted therapeutic approaches. Flavonoids, abundant in fruits, vegetables, and medicinal plants, have demonstrated significant potential in counteracting key pathological processes underlying sarcopenia. Compounds such as quercetin, kaempferol, and rutin inhibit inflammatory pathways, regulate oxidative stress, enhance mitochondrial biogenesis, and promote the anabolic signaling essential for muscle maintenance and regeneration. Emerging evidence from in vitro and in vivo models further supports the beneficial effects of baicalin, genkwanin, isoschaftoside, naringin, eriocitrin, and puerarin on muscle preservation and recovery (Figure 2).

Importantly, while many of these effects have been demonstrated in preclinical settings, their clinical translation remains an active area of investigation. The diagnostic framework proposed by the EGWSOP2—which emphasizes muscle strength, quantity, and physical performance—provides a useful benchmark to evaluate therapeutic outcomes. Notably, some compounds such as rutin have shown promise in human studies, improving muscle function when combined with protein supplementation. These findings suggest that flavonoids may complement current preventive strategies, such as resistance exercise and nutritional support, particularly in elderly individuals at risk of sarcopenia. However, further human trials are needed to validate their efficacy based on standardized diagnostic criteria and to determine optimal dosage, bioavailability, and long-term safety.

Compared to traditional therapies such as rapamycin, myostatin inhibitors, or hormone-based interventions, flavonoids offer distinct advantages including low toxicity, pleiotropic effects, and availability from natural sources. While agents like rapamycin target mTOR signaling to promote autophagy, and myostatin antibodies inhibit catabolic signaling, these approaches may present immunosuppressive or metabolic side effects. Flavonoids, in contrast, simultaneously modulate inflammation, oxidative stress, mitochondrial function, and anabolic pathways with favorable safety profiles. However, limitations such as poor oral bioavailability and variable systemic absorption remain obstacles to clinical translation. Thus, understanding the pharmacological properties and optimization strategies of flavonoids is essential to complement or improve upon current therapies.

A critical consideration for clinical applicability is the dosage required to achieve therapeutic efficacy. In preclinical studies, effective concentrations of flavonoids often range from 10 to 100 mg/kg/day in rodents or 10–50 μM in cell culture models. When translated to human equivalent doses, these correspond to approximately 300–800 mg/day for a 60 kg adult—levels that far exceed typical dietary intake, which is generally below 100 mg/day for total flavonoids and 10–20 mg/day for quercetin. Thus, while flavonoids are accessible through fruits and vegetables, pharmacologically relevant doses are unlikely to be achieved through diet alone. This highlights the potential role of supplementation, bioavailability-enhanced formulations, or synergistic combinations to meet therapeutic thresholds.

From a structure-activity perspective, the presence and position of hydroxyl groups, the degree of methoxylation, and especially glycosylation critically influence the bioactivity and bioavailability of flavonoids. For example, quercetin glycosides such as rutin and isoquercitrin exhibit higher water solubility and enhanced intestinal absorption compared to aglycone quercetin, although the latter may exert stronger direct biological effects in vitro. These modifications not only affect plasma stability and transport but also determine tissue-specific uptake. Importantly, glycosylation may also facilitate interactions with transporters and metabolizing enzymes, ultimately influencing systemic distribution and efficacy in muscle tissue.

Moreover, recent research suggests that sarcopenia is not solely a muscle-localized condition but involves cross-talk between skeletal muscle and other organs, including adipose tissue, liver, and immune cells. Flavonoids capable of modulating systemic inflammation, gut microbiota, or metabolic status (e.g., resveratrol, puerarin) may thus provide multi-organ benefits relevant to sarcopenia. This integrated view underscores the potential of flavonoids as systemic modulators of aging physiology, rather than localized agents.

Although this manuscript includes a summary table listing the effective doses used across studies, the dose–response relationship remains poorly characterized in most reports. Few studies directly compare different doses of flavonoids on muscle outcomes in a systematic manner. Addressing this gap will be crucial for establishing safe and effective dosing regimens, and for understanding whether therapeutic effects follow linear or threshold-based kinetics. Therefore, future research should prioritize structured dose-response analyses and pharmacokinetic profiling in both animal and human models.

While this review focuses on the mechanistic effects of individual flavonoids, it is important to consider that flavonoids often coexist in natural food matrices and may exert synergistic or additive effects when administered in combination. Several studies have reported that combining flavonoids such as quercetin and kaempferol [139], or resveratrol and catechins [140], can enhance anti-inflammatory and antioxidant responses more effectively than single compounds alone. However, studies specifically investigating whether such flavonoid combinations synergistically alleviate sarcopenia remain limited. This represents a promising area for future research.

In addition to the effects of individual flavonoids on muscle preservation, recent studies have explored senolytic strategies targeting cellular senescence—a key contributor to age-related tissue dysfunction. Agents such as quercetin combined with Dasatinib, fisetin, and resveratrol have demonstrated a capacity to selectively eliminate senescent cells and attenuate systemic inflammation in aging models. These senolytics have shown potential to improve muscle mass, reduce SASP factors, and restore regenerative capacity, highlighting a novel therapeutic avenue for sarcopenia [141,142,143,144]. However, the flavonoids highlighted in this review have not been directly confirmed to exhibit senolytic effects in sarcopenia, and further investigation is needed to elucidate their potential in targeting senescent cells in skeletal muscle.

Despite promising preclinical results, clinical studies remain limited. Future research should emphasize dose-finding trials, mechanistic investigations on effective plasma concentrations, and the integration of pharmacokinetic data to guide translational development. Harnessing the bioactive potential of these natural compounds offers an attractive and accessible strategy to mitigate age-related muscle loss and improve quality of life in aging populations.

## Figures and Tables

**Table 1:** Flavonoids against sarcopenia.

Compound	Model	Molecular Targets	Effects	Dose	References
Quercetin	C2C12 cells and C57BL/6 mice	ERK↓ p38↓ MAPK↓ NF-κB↓ Atrogin-1↓ MuRF1↓	Synthesis and differentiation of muscle cells and reduction of inflammation	50 μM 0.05% or 0.1% in diet	[22]
	C2C12 cells and C57BL/6 mice	Atrogin-1↓ MuRF1↓ HO1↑ Nrf2↑ NF-κB↓	Inhibition of TNFα-induced inflammatory response and muscle protein degradation	20–50 μM 0.05% in diet	[23]
	C2C12 cells and BALB/c CrSlc mice	Atrogin-1↓ MuRF1↓ Foxo1↓	Protection of muscle atrophy induced by dexamethasone	10 μM 0.15% or 0.45% in water	[24]
	Primary mouse myoblast	PPAR-γ↓ FABP4↓ MyoD↓ Pax↓	Inhibition of muscle adipogenesis	5–50 μM	[25]
	Primary human muscle cell	CEBPA↓ ADIPOQ↓	Inhibition of muscle adipogenesis	0.3-3 μM	[26]
	BALB/c mice	PPAR-δ↑ CPT1↑ HADH↑ UCP3↑	Promotion of glycogen storage and fatty-acid oxidation	0.005% in diet	[27]
Rutin	Litopenaeus vannamei (shrimp)	Enolase↑ malate dehydrogenase↑ ATP/ADP translocase↑ EF-1α↑ elF2α↑ aspartate aminotransferase↑	Protection of protein deterioration induced by T-2 toxin	2.00–32.00 g/kg in diet	[28]
	ICR mice	phosphorylation of AMPK↑ CAMKKβ↑ ACC↑	Promotion of glucose uptake and prevent hyperglycemia in high-fat diet obese mice	0.1% in diet	[29,30]
	Wistar rats	Calcium uptake↑ CAMKII↑ GLUT-4 translocation↑	Promotion of glucose uptake by insulin-independent calcium pathway	10^−14^ M	[31]
	Sprague-Dawley rats	AMPK↑ mtDNA↑ Atrogin-1↓ MuRF1↓ PGC-1α↓ Nrf1↓ Bax↓ Bcl2↓	Promotion of mitochondrial biogenesis in high-fat diet obese rats and inhibits muscle cell apoptosis	50 mg/kg/day	[32]
	Sprague-Dawley rats	PGC-1α↑ NRF1↑ Tfam↑ SIRT1↑	Promotion of mitochondrial biogenesis in high-fat diet obese rats	0.1% in diet	[32]
	C57BL/6 mice	MyoD↑ MyoG↑ MHC↑	Protection of muscle atrophy induced by dexamethasone	0.1% of extract	[33]
	C2C12 cells	IL-6↓ TNFα↓ iNOS↓ ROS↓ NF-κB↓	Inhibition of inflammation induced by LPS	10–100 μM	[34]
	C2C12 cells	MAFbx↓ MuRF1↓ FOXO3↓	Protection of muscle atrophy induced by dexamethasone	100 μM	[35]
	VSMC of C57BL/6 mice and chickens and broilers	malondialdehyde↓ ROS↓ 3-nitrotyrosine↓ 4-hydroxynonenal↓ p47phox↓ Nox4↓	Protection from hydrogenperoxide-induced senescence	40 mg/kg/d	[36,37,38]
	Brioiler chickens	LC3-II↓ PINK1/Parkin↓	Increased antioxidant activity and inhibited mitophagy	500 mg/kg	[38]
	Sqiss mice	MDA↓ BUN↓ lactate↓ GOT↓ GPT↓ nNOS↓ iNOS↓ TNFα↓ syncytin-1↓ phosphorylation of IKK↓	Anti-oxidant activity in high-fat diet obese mice, increasing running ability	100–200 mg/kg/day	[39,40]
Kaempferol glycosides	Wistar rats	PI3K↑ GSK-3↑ MAPK↑ PP1↑	Promotion of glucose uptake and glycogen synthesis in soleus muscle	1 μM	[41]
	L6 skeletal muscle from rat	SIRT1↑ p-IRS↑ AKT↑ AMPK↑, GLUT4 translocation	Enhancement of glucose uptake	100 nM or 1 μM	[42]
	HSMM and DHSMM	CAT↑ MDA↓	Anti-oxidant activity combined with metformin	400 µg/mL of extract	[43]
	Broiler chickens	TBARS↓ MDA↓	Inhibition of oxidative stress via reduced MDA concentration	0.3 or 0.6% in diet	[44]
	Wistar rats	Oxidative fibers↓	Attenuation of muscle atrophy in cerebral palsy rats	1 mg/kg	[45]
Baicalin	BALB/c mice	Atrogin-1↓ MuRF1↓ IκBα↓ p65↓ NF-κB↓	Inhibition of muscle atrophy in cachexia mice	50 or 150 mg/kg	[46]
Genkwanin	C2C12 cells	2-NBDG↑	Enhancement of glucose uptake	50 µg/mL extract	[47]
	C57BL/6 mice	MyHC-II↑ Myh4↑ Foxo1↓ Foxo6↓ IL-1β↓ STAT3↓ p65↓	Anti-inflammation and inhibition of osteosarcopenia in geriatric mice	500 mg/kg/d extract	[48]
Isoshaftoside	C2C12 cells	Myf5↑ Myf6↑ MyoD↑ Myogenin↑ TNFα↓ IL-1α↓ IL-1β↓ IL-6↓	Prevention of muscle degradation and promotes muscle regeneration Induction of mitochondrial biogenesis Reduction of inflammation	50 μM	[49]
Naringin	BALB/c mice	SOD↑ CAT↑ GSH-Px↑ ROS↓	Reduction of fatigue and enhanced endurance Reduced oxidative stress	400 mg/kg in diet	[50]
	Obese rats	mTOR↑ PGC1-α↑ Atrogin-1↓ MuRF1↓ SOD1↑ CAT↑ ROS↓	Reduction of protein degradation and increased protein synthesis Induction of mitochondrial biogenesis	50 and 100 mg/kg	[51]
Eriocitrin	denervated mice	Atrogin-1↓ MuRF1↓	Reduction of oxidative stress and inhibits lipid peroxication	0.5% in diet	[52]
Puerarin	Type 1 diabetic rat	Akt/mTOR↑ PGC-1α↑ AMPK↑	Enhancement of mitochondrial biogenesis Prevents skeletal muscle wasting	100 mg/kg/d	[53,54]
	L6 myotubes			0.3 mM	

↓ indicates decrease in expression and ↑ indicates increase in expression.

## Abbreviations

2-NBDG	2-(N-(7-Nitrobenz-2-oxa-1,3-diazol-4-yl)Amino)-2-deoxyglucose
ACC	Acetyl-CoA Carboxylase
ADIPOQ	Adiponectin
AKT	Protein Kinase B
AMPK	AMP-activated Protein Kinase
ATP/ADP translocase	Adenine Nucleotide Translocator
Atrogin-1	Muscle Atrophy F-box Protein
BUN	Blood Urea Nitrogen
Bax	Bcl-2-associated X Protein
Bcl2	B-cell lymphoma 2
CAMKII	Calcium/Calmodulin-dependent Protein Kinase II
CAMKKβ	Calcium/Calmodulin-dependent Protein Kinase Kinase Beta
CAT	Catalase
CEBPA	CCAAT/Enhancer-binding Protein Alpha
CPT1	Carnitine Palmitoyltransferase 1
EF-1α	Elongation factor 1-alpha
eIF2α	eukaryotic translation initiation factor 2 subunit alpha
ERK	Extracellular Signal-Regulated Kinase
Elongation factor 1-alpha	Translation Elongation Factor 1 Alpha
FABP4	Fatty Acid Binding Protein 4
FOXO3	Forkhead Box O3
Foxo1	Forkhead Box O1
Foxo6	Forkhead Box O6
GLUT4	Glucose Transporter Type 4
GOT	Glutamate Oxaloacetate Transaminase
GPT	Glutamate Pyruvate Transaminase
GSH-Px	Glutathione Peroxidase
GSK-3	Glycogen Synthase Kinase-3
HO1	Heme Oxygenase 1
IKK	Inhibitor of κB Kinase
IL-1α	Interleukin-1 Alpha
IL-1β	Interleukin-1 Beta
IL-6	Interleukin-6
IκBα	Inhibitor of Nuclear Factor Kappa-B Alpha
LC3-II	Microtubule-associated Proteins 1A/1B Light Chain 3B
MAFbx	Muscle Atrophy F-box Protein
MAPK	Mitogen-Activated Protein Kinase
MDA	Malondialdehyde
MHC	Myosin Heavy Chain
MuRF1	Muscle RING-finger Protein-1
MyHC-II	Myosin Heavy Chain II
Myf5	Myogenic Factor 5
Myf6	Myogenic Factor 6
Myh4	Myosin Heavy Chain 4
MyoD	Myogenic Differentiation 1
MyoG	Myogenin
Myogenin	Myogenic Regulatory Factor
NF-κB	Nuclear Factor Kappa-light-chain-enhancer of Activated B Cells
NRF1	Nuclear Respiratory Factor 1
Nox4	NADPH Oxidase 4
Nrf1	Nuclear Respiratory Factor 1
Nrf2	Nuclear Factor Erythroid 2-related Factor 2
PGC-1α	Peroxisome Proliferator-Activated Receptor Gamma Coactivator 1-Alpha
PI3K	Phosphoinositide 3-Kinase
PINK1/Parkin	PTEN-induced Kinase 1/Parkin RBR E3 Ubiquitin Protein Ligase
PP1	Protein Phosphatase 1
PPAR-γ	Peroxisome Proliferator-Activated Receptor Gamma
PPAR-δ	Peroxisome Proliferator-Activated Receptor Delta
Pax	Paired Box Protein
ROS	Reactive Oxygen Species
SIRT1	Sirtuin 1
SOD1	Superoxide Dismutase 1
STAT3	Signal Transducer and Activator of Transcription 3
TBARS	Thiobarbituric Acid Reactive Substances
TNFα	Tumor Necrosis Factor Alpha
Tfam	Transcription Factor A, Mitochondrial
iNOS	Inducible Nitric Oxide Synthase
nNOS	Neuronal Nitric Oxide Synthase
